# On the Prolapsus Ani in Grown Persons
*Medico-Chirurgical Illustrations, &c.


**Published:** 1831

**Authors:** 


### On the Prolapsus Ani in Grown Persons.*

This, as Mr. Fletcher well remarks, is a disease on which less has been written than might have been written, and much less is known than ought to be known. To supply the former deficiency, and the latter desideratum is the object of his chapter on the subject. We need scarcely inform our readers that Mr.

* Medico-Chirurgical Illustrations, &c.

Hey and Mr. Copland have severally proposed and performed operations for the prolapsus ani. That of the former consisted in the removal, by a circular incision, of the pendulous flap and other protuberances surrounding the anus; that of the latter, in pinching up a fold of the lining membrane of the rectum within the gut, and tying a ligature around it sufficiently tight to procure its sloughing. From this procedure there is a risk of inflammation of the gut, independent of which Mr. Fletcher understands that the return of the complaint after its performance is a frequent occurrence. The external, or Mr. Hey's operation, is occasionally, but very rarely followed by hæmorrhage. In fifty cases Mr. Fletcher has seen this occur once.

"Mr. Hey, and others too, who operated exactly after his manner, had occasionally inflammatory symptoms succeed the operation. This must have been the effect of cutting away without scruple, and often deeply and roughly, the little blue tumors at the verge of the anus, many of which were formed by the extremity of the anus itself. There is no occasion for this to be done; the gut ought not to be unnecessarily cut, and in this point Mr. Hey's operation may be improved. A deliberate dissection will always be capable of separating the loose skin which you require for excision, from the bowel itself, which should be tenderly and carefully returned. Out of the fifty cases in which I have operated, I have not seen one where the operation was followed by inflammatory symptoms, nor (with one exception,) where it did not succeed in entirely curing the prolapsus."

To illustrate his opinions and exemplify his practice, Mr. Fletcher relates the particulars of eight cases. We will select the most important.

CASE 1.—*Prolapsus Ani in its simplest form.* "A gentleman, about fifty years of age, when taking exercise in walking, would have the bowel descend to the extent of two or three inches beyond the verge of the anus, and also after a stool. Some bleeding would then take place from its surface, the effects of the squeezing of the sphincters. The pain on these occasions was so intolerably severe, as to oblige him, even in the field, to lie down instantly upon his back, and reduce the protrusion, which he could always effect, by gentle pressure. The repetition of his sufferings in this way, for years, at last led him to his surgeon. By straining, as at stool, at my request, the gut was brought into view, in folds, forming a tumour about the size of a small lemon, surrounded at its base by several loose projections of purple-looking integuments, which the gut, in its descent, had brought more completely into view than it could have been before such descent took place. He was now in dreadful pain; the bowel was of a dark red, or purple tone of colour, from the pressure of the surrounding sphincter. From this circumstance, together with the fact, that the bowel never descended on rising out of bed in the morning, and only after much straining at the water-closet, or in walking exercise, I suspected that this muscle was still strong, but not sufficiently so to prevent the gut passing through it.

On reducing the prolapsed part, this was found to be the case. The sphincter, irritated by the protruded part, which had just re-ascended, still acted sharply upon my fingers, now engaged in an examination of the interior of the gut."

An operation was proposed and assented to. The patient leaned with his belly over the edge of a high table. The gut was now brought down by straining, with the folds of loose skin more apparent. It was replaced sufficiently to keep it clear of the knife, and a full sized rectum-bougie passed a little way into its canal, but not so far as to carry back the folds of loose skin at all out of the view of the operator. The bougie of a full size is useful on this occasion, for as the patient should have to bear down, to bring the whole of the loose skin outside the anus, the gut would descend at the same moment, were it not for the instrument. I selected five of these portions of integument, passing a thread through each, and drawing these in succession with some force along the bougie which kept the gut back, they were excised very close to the plane of the surrounding parts. The wound was well washed with cold water, the gut completely restored to its natural position, and an appropriate compress and bandage applied to support it there, as the tendency to its descent is always increased by the operation, until the excisions are contracted and healed. A pupil was left in attendance, to see that there was no hemorrhage, to which this operation is rather liable, and of which nearly a fatal example will be found in a succeeding case. In the evening the patient complained of violent pain. On examination, the gut had descended in his attempt to pass air. The pain was doubtless the effect of the pressure of the sphincter. The gut was replaced with a globular compress fixed over the site of the anus, with an injunction to the patient, to restrain his inclination to pass wind.

The contraction of the anus in this case was so great during cicatrization, as to occasion inconvenience in the passage of the stools. This was remedied by a bougie, and for some years the patient has been entirely cured. It is better in these cases to open the bowels on the day preceding the operation, and to give no solid food on that day.

Case 2. *Prolapsus Ani—Power of the Sphincter extinct—Nearly fatal Bleeding from the Operation.*

A lady consulted our author for a prolapsus of the bowels of immense size; it was almost constantly down, and would descend on her standing, even for a moment. The lady was old, and relaxed; the sphincter exceedingly lax. She had indulged greatly in purgative medicines. There was very little loose integument about the verge of the anus, but an operation was desired and therefore performed. Considerable portions of slightly projecting skin were raised from the inner margin of the anus, and a wound nearly surrounding it was left. A compress and bandage were applied, and for some time a gentleman sat with the patient. About three hours after the operation Mr. Fletcher was summoned, and found her almost in articulo mortis, from profuse hæmorrhage. Brandy, hot bottles, &c. were in requisition, the patient rallied, a small vessel was discovered bleeding on the right side of the anus, it was secured, and she recovered. After the healing of the wounds, the bowel descended to the extent, perhaps, of one-fifth of its former size, when the patient was at the water-closet. With the aid of a spring truss, with a spherical pad, the prolapsus was afterwards tolerably manageable.

Case 3. *Prolapsus Ani, with extensive Adhesion to the surrounding Skin—Operation successful.*

Mrs. ——, æt. 44, had had prolapsus ani irreducible for four years. The bowel being in concentric folds, and was adherent in several points to the skin, whilst the sphincter was exceedingly relaxed. "After clearing the bowels well on the previous day, and permitting the patient to take slops only of gruel and milk, I proceeded to the operation, endeavouring first to remove the gut out of the way, by reducing it, and then restraining its descent by the introduction of a large bougie, whilst I dissected the skin freely away, by the side of the instrument, from the anus and bowel. But still the gut would descend, or was pulled down by the adhesions by the side of the bougie in places, and I therefore ultimately had the intestine held aside by the assistant, whilst those adhesions were separated, and all the loose skin freely removed, which was pulled out forcibly from the anus with a tenaculum. It was curious to see, as soon as the incisions were completed, the muscular coat of the intestine, together with the relaxed sphincter, begin to act vigorously, and carry the gut into its proper place, and retain it there with ease, whilst all my attempts to accomplish this with my own fingers were unavailing. The stimulus of pain, coupled with the liberation of the adhesions, was probably the source of this renovated vigour.

When it is desirable to make the adhesive process as firm and as contracted as possible, I am in the habit of laying pieces of sponge upon the anus, and filling the hollow of the buttock with it, so that considerable pressure is made upon the part, which is already contracted or tucked up: the muscular action is assisted in retaining that tucked up character by the external pressure."

The lady was kept in the recumbent posture, and recovered with the anus as smooth as ever, though the aperture required, from its small size, occasional enlargement with a candle.

*Case* 4. Is that of an elderly maiden lady who had a tumour about the size of a double walnut, which proved to be the lowest portion of the bowel adherent to a portion of loose skin just within the anus. The operation consisted in separating, by dissection, the fold of skin from the intestine, to an extent sufficient to ensure union to the sphincter. The operation prevented any further descent, and the remains of the tumour disappeared by absorption within the gut. The next case was one of old prolapsus ani, with a cartilaginous tumour, about the size of a large garden bean, hanging by a long pedicle from deep within the verge of the anus. Mr. F. cut away the small tumour at the farthest end of its pedicle, and then, introducing a bougie into the rectum, operated for the prolapsus in the manner already noticed. On her discharge from the hospital there was a very slight appearance of the gut when she strained on the side of the anus, where a bit of loose skin had been left.

The preceding cases were given with the view of shewing the necessity of ascertaining, as far as possible, the causes of prolapsus ani, before attempting its radical cure by operation. In order to ascertain whether a stricture exists in the rectum, Mr. F. recommends that a bougie, previously softened in warm water and adapted to the curves of the gut, should be passed its whole length. A stricture is capable of occasioning prolapsus ani, by the great efforts which it causes the patient to make. With respect to Mr. Fletcher's recommendation respecting the use of the bougie, we cannot forbear remarking, first, that it is contrary to the advice of some of our best surgeons; secondly, that if followed by inexperienced persons it might be productive, as undoubtedly it has been, of disastrous consequences. Ordinary stricture of the rectum is seated at no great distance from the anus; the stricture at or about the sigmoid flexure of the colon is hardly amenable to the bougie without a considerable degree of risk. But this by the way.

CASE 6. *Prolapsus Ani, conjoined with Stricture of the Rectum.*

Miss ———, æt. 25, consulted our author for piles, from which she said she had suffered for nine or ten years. She was emaciated, and very nervous. She said that something would come down after every evacuation, but would return after she had lain on the bed for an hour or two, when burning pain occurred, and continued about the anus and lower part of the back. On attempting to walk the bowel descended, the stools were passed with great difficulty, and were remarkably small. On examination, a very large prolapsus of the lower extremity of the bowel took place when the patient strained. On replacing the prolapsus and introducing the finger, a stricture was found at about an inch and a half from the anus, and the gut was found contracted, exquisitely tender, and thickened, as far as the finger could reach. She had been subject to difficulty in passing her motions, and burning pains for many years—to prolapsus for three years.

Under these circumstances our author imagined that the prolapsus was the consequence of the stricture, and gave a cautious prognosis. The bougie was carefully employed for four months, at the end of which time a full sized one could be passed nearly 11 inches. The operation for prolapsus ani was then performed, succeeded well, and the patient is now a fine healthy young woman.

"The foregoing is a very serious example of the folly and mischief arising out of the practice of prescribing for *supposed* complaints, the product of the patient's own judgment or imagination, the real nature of which might have been readily discovered by a proper examination of the parts concerned. No examination was ever made, in this case, until the lady came under my observation; and even then she said her malady was piles; and they all say so. All the various affections of the anus are so called by the patients. But the medical man ought not to copy the errors of his patient, to believe without evidence or conviction, which will assuredly lead him to prescribe for diseases which exist only in their conjoint imaginations.

Here was a young lady who lost many of the best years of her life, and what was worse, spent them in wretched suffering, or in swallowing loads of useless medicine; nay, further, who was carried into the very jaws of death, by one complaint of a very trifling nature being mistaken for another of the highest importance to comfort, and to life itself.

Had an early examination of the parts concerned been carried into effect, the true affection would have been quickly revealed, and the sad miseries she subsequently endured, wholly prevented. When a discovery of the real malady was at length made, she was cured, without any medicine, in fewer months than years had been previously occupied in pursuing a wrong course.

This case then will furnish another valuable lesson, which indeed may daily be taught, if we would learn, viz.—never to prescribe for affections of the anus, without a proper visual and manual enquiry into their real character."

In the next case, a lady was operated on for prolapsus ani by the ligature. The procidentia was benefited, but she suffered, and, anterior to the operation, had suffered from costiveness, pains in the loins, &c. On examination, Mr. Fletcher found a stricture of the rectum at the distance of five inches from the anus. After a time a bougie was got through the stricture, and a recovery is anticipated by Mr. F.

Case 8. *Prolapsus from Stricture—temporary Cure from Operation—Fistula Ani.*

A lady, æt. 50, the mother of many children, had undergone excision for prolapsus ani with temporary benefit. Soon, however, a difficulty of voiding her stools, which had previously existed, was increased, with a deceptive feeling of protrusion. Then an abscess formed by the side of the rectum, and the gut was found to descend occasionally within the painful gripe of the sphincter, but never to pass through it. There were also some symptoms of stricture. The abscess was laid open into the gut, and some portions of loose skin were removed. Before the wound was healed, a stricture was discovered at four inches and a half from the anus. Though a bougie of moderate size is passed three times a week the stricture is not wholly removed. No recurrence of the procidentia has hitherto taken place.

Our author concludes by the mention of another case, in which there is prolapsus combined with stricture at five inches from the anus; no bougie can be got through it. With the following brief observations we conclude.

"In my own practice, the prolapsus ani has occurred much more frequently in females than in males, in the proportion of seven to one; a fact that may probably be accounted for on the ground of their relaxed texture, and indulgence in physic, which must ever weaken and relax the parts concerned in this very distressing and disgusting complaint."

"Writers on stricture of the rectum may have mentioned prolapsus as an occasional effect of it. But I do not remember any writer on the prolapsus ani itself who has considered stricture as one of its causes, and thence derived a rule to examine the whole extent of the bowel, previous to any operation for the cure of the first affection."

Perhaps none of the minor operations of surgery require more tact and experience than this examination of the whole course of the rectum with the bougie. The natural obstructions in the canal are many, and may be still more numerous from disease and other circumstances; and such difficulties can only be met successfully by a knowledge of the anatomy, natural and morbid.

The angles made by the bowel itself, the projection of the sacrum, an enlarged or retroverted uterus, or prostate gland, a spasmodic stricture, or hardened fæces, are all to be taken into consideration, and well remembered by the surgeon, otherwise he may take some of these obstructions to the passage of the bougie for a stricture, when, in reality, no such affection exists. The character of the bougie itself may increase the difficulties.

If it be too small, or too soft, these natural obstructions to its route will turn back the point, and leave it curved in the bowel, thus confirming the original error."

We think the cases worthy of the attention of practical surgeons.

